# Anesthesia for a Patient With Cerebrovascular Accident with Seizure Disorder Undergoing Medical Termination of Pregnancy: A Case Report

**DOI:** 10.7759/cureus.42487

**Published:** 2023-07-26

**Authors:** Saiesh Dessai, Sanjot Ninave, Amol Bele, Shiras P

**Affiliations:** 1 Department of Anaesthesiology, Jawaharlal Nehru Medical College,Datta Meghe Institute of Higher Education and Research, Wardha, IND

**Keywords:** cerebrovascular accident (stroke), multi-focal seizure, refractory seizure, ketamine, propofol, procedural sedation and analgesia, total intravenous anesthesia (tiva)

## Abstract

The term “total intravenous anesthesia” refers to the preservation of an anesthetic plane with the use of an injectable anesthetic, a sedative that is often given in intermittent boluses. The tendency to have recurrent unprovoked seizures is known as epilepsy. Its prevalence ranges from 0.5% to 1%. The highest incidence rates are in those with anatomical or developmental brain abnormalities, as well as at the extremes of age. The most common triggering factors for epilepsy are stress and fear. The main advantage of total intravenous anesthesia is the patient's rapid recovery and early ambulatory. Conscious sedation is a strategy for giving patients excellent anesthesia and analgesia. In this case report, we will describe a high-risk epileptic patient who required conscious sedation to perform a medical termination.

## Introduction

Total intravenous anesthesia (TIVA) refers to the preservation of an anesthetic plane with the use of an injectable anesthetic, a sedative [1-3]. After stroke, epilepsy is the second most prevalent neurological condition. In affluent nations, the age-adjusted incidence is 24-53 per 100,000 person-years. The highest incidence rates are in those with anatomical or developmental brain abnormalities, as well as at the extremes of age [4]. Males and those in lower socioeconomic classes are more likely to experience it. Epilepsy affects 5.59 people out of every 1,000 people in India [5]. The management of an epileptic patient during the intraoperative phase presents a significant challenge to the attending anesthesiologist. Anesthesiologists may have to deal with a variety of pharmacological interactions between anesthetics and anti-epileptics, manage seizures during and after surgery, and manage status epilepticus, to name a few [6]. It has been employed in several surgical procedures and has also been investigated for its potential use in seizure control. Refractory status epilepticus (RSE) may be treated safely and effectively with TIVA, which can be difficult to manage with standard medication [7]. This report focuses on a specific case where anesthesia is administered to a patient with a history of cerebrovascular accident (CVA) and seizure disorder during a medical termination of pregnancy. Each of these elements adds its own set of challenges and potential risks including the anesthetic agents and drugs, which can potentiate seizures. This report will highlight the successful management and anesthetic technique used in this case.

## Case presentation

**Figure 1:**
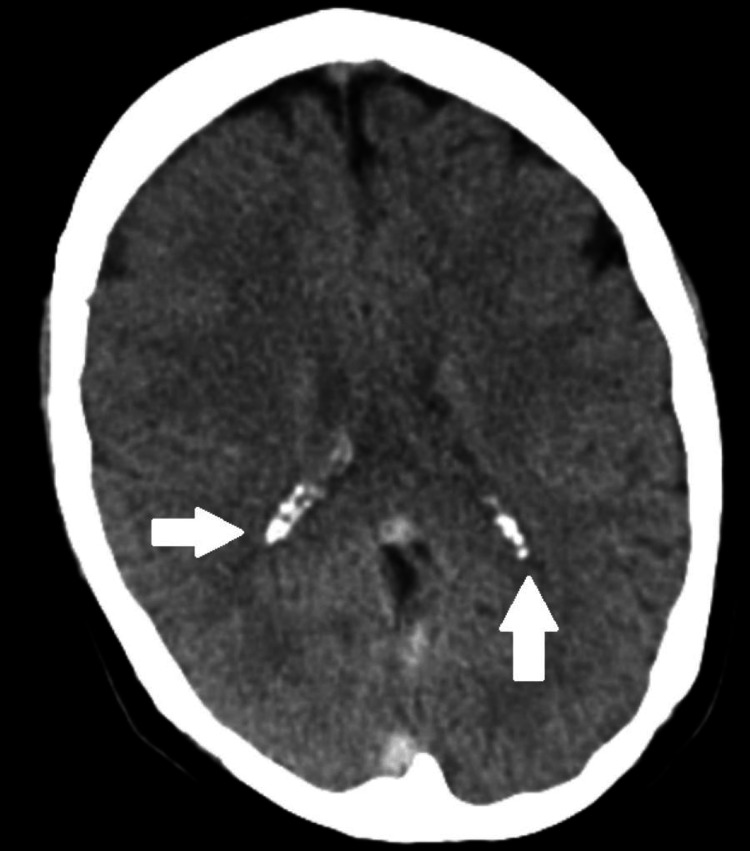
Computed tomography scan of the brain showing venous thrombosis

**Figure 2:**
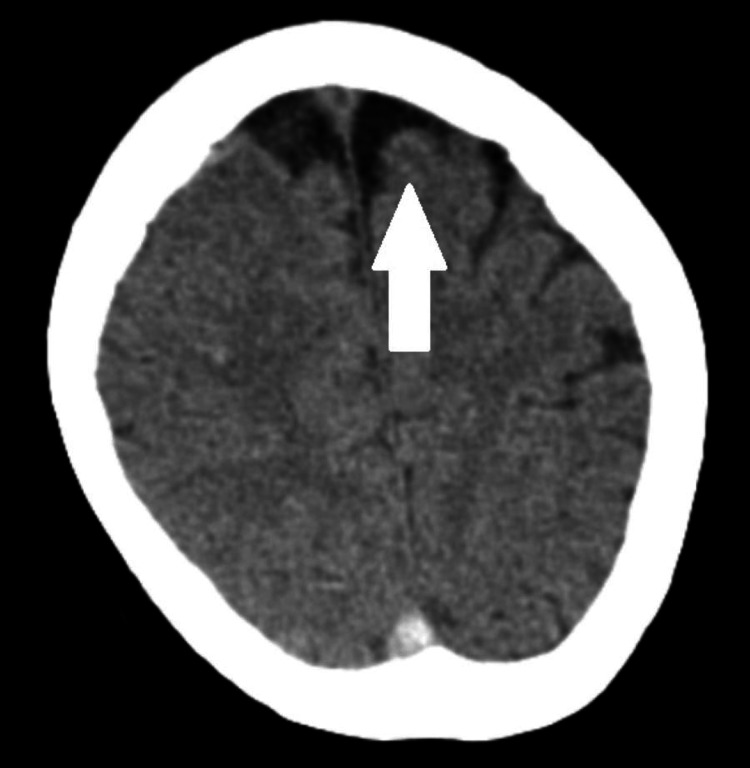
Computed tomography scan showing ill-defined area of hypodensity

A 30-year-old female patient presented with a history of amenorrhea for nine weeks and was planned for medical termination of pregnancy. Her obstetric was gravida 2 para 1 living 1; her gestation period was 8 weeks. The patient’s past medical history was significant for a varicose vein in the bilateral lower limb, for which she underwent surgery five months ago under a subarachnoid episode. In June 2022, the patient was admitted because of weakness in the right upper and lower limb with an episode of generalized seizure. On admission, a computed tomography scan of the brain revealed vertebral venous thrombosis in the superior, inferior, sagittal straight sinuses, right transverse, and sigmoid sinuses (Figure 1). An ill-defined area of hypodensity in the right frontal region is most likely to be a venous infarct (Figure 2). Thrombosis in the superficial cortical vein in the bilateral parietal region is also seen. Her routine blood investigations were conducted, which showed the following: hemoglobin level of 7 g/dL, total white blood cell count of 7,200/mm^3^, platelet count of 216 x 1000/mm^3^, international normalized ratio of 1.21, thyroid-stimulating hormone level of 5.8 mIU/ml. The patient was started on an injection of enoxaparin 60 mL subcutaneously, an injection of level 500 mg, an injection of mannitol 100 mg IV, and symptomatic treatment. There was no history of ventilator support. On discharge, the patient was started on oral dabigatran 150 mg, oral levetiracetam 500 mg, and tablet rivaroxaban 15 mg non-compliant. Hypothyroidism was diagnosed on admission and was started on tablet thyroxin 100 μg.

On her admission for medical termination of pregnancy, the patient was off rivaroxaban for five days. On examination, the patient was vitally stable. Airway assessment mouth opening was three fingers with Mallampati grade 2. The patient's physical condition was evaluated per ASA (American Society of Anesthesiologists) physical status 3. On examination, her pulse rate was 90 beats per minute and a blood pressure was 124/82 mmHg. Power was normal in all four limbs without any neuro- or sensory deficits. The patient was advised nil per oral for 8 hours, premedicated with tablet pantoprazole 40 mg, and instructed to take an anti-thyroid and anti-epileptic medication on the day of the procedure. Considering the surgery period and previous medical history, the patient was planned for surgery under TIVA. The left dorsal vein was cannulated using a 20 g cannula. Before the start of the procedure, premedication was given, which included an injection of glycopyrrolate 0.2 mg IV, an injection of midazolam 1 mg IV, an injection of butrum 1 mg IV, an injection of propofol 140 mg in divided doses (over 15 minutes), and an injection of levetiracetam 250 mg IV at the start of surgery. Supplemental oxygen was provided via a face mask at 6 liters. The patient was observed using a non-invasive blood pressure monitor, mainstream end-tidal carbon dioxide, and a pulse oximeter throughout the procedure. Her initial vitals were as follows: blood pressure of 118/72 mmHg, pulse rate of 86 beats per minute, and oxygen saturation of 100%. Throughout the procedure, hemodynamic stability was preserved. Additionally, paracetamol was infused to alleviate post-operative discomfort. The procedure lasted for 15 minutes and was uneventful. The patient was observed for 10 minutes post-surgery for signs of seizures, altered respiratory pattern, and changes in oxygen saturation, and was shifted to post-operative care ward for observation for 24 hours.

## Discussion

**Figure 3:**
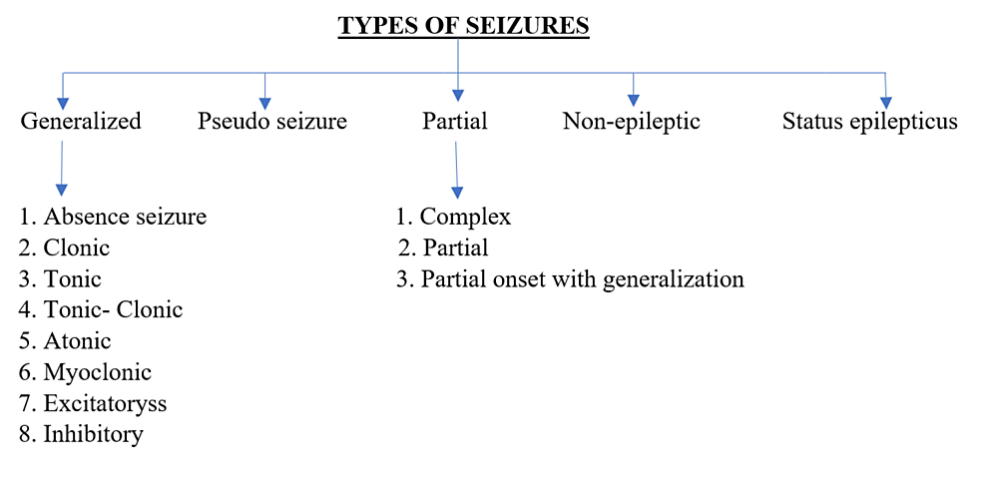
Types of seizures

The most effective way to utilize TIVA in monitored anesthesia treatment is in combination with a dense field block or nerve block. To ensure that patients are comfortable enough for their surgery while preventing airway blockage, midazolam, fentanyl, propofol, and ketamine can be utilized alone or in a balanced strategy in monitored anesthesia instances [3,8]. Drugs such as ketamine, etomidate, and methohexital are proconvulsant and anti-convulsant, depending on dosage [9] . Numerous reports of sevoflurane-induced seizures, particularly in children, have been documented [4]. The opioid having the highest correlation to myoclonus and tonic-clonic seizure activity is meperidine. However, it has been noted that low-to-moderate doses of fentanyl, alfentanil, sufentanil, and morphine, particularly after intrathecal use, can result in generalized seizure patients [4]. Propofol, thiopental, methohexital, and pentobarbital are widely known for treating RSE [10]. All agents work as anti-convulsants at higher dosages [4]. Ketamine functions as a glutamate antagonist at NMDA receptors. Low doses of ketamine, like the other intravenous medications, could exacerbate seizure activity. However, at higher doses, it has anti-seizure effects, which may be helpful in the treatment of resistant status epilepticus [11]. Seizure is classified into five types (Figure 3).

Managing an epileptic patient is challenging for the anesthesiologist, who must take several skilled and informed steps to accomplish a safe anesthetic treatment [6]. Preoperative, perioperative, and post-operative concerns for managing anesthesia in patients with epilepsy are extensive. Anesthesia assessment and a regular pre-anesthetic examination should check for related seizure disorders that may be caused by conditions such as tuberous sclerosis, Huntington's chorea, neurofibromatosis, or Lesch-Nyhan syndrome [6]. Similarly to this, it is essential to take into account any potential anesthetic and anti-epileptic medication interactions since hepatic enzyme stimulation requires the use of higher doses of opioids. Pre-anesthetic checkup should include thorough clinical history, and examination should also check for any potential adverse effects of continued epileptic medications, such as megaloblastic anemia and gingival hypertrophy. Typically, patients are advised to continue anti-convulsants on the day of surgery, depending on the usual medication intake timing. Sometimes it is recommended that the patient stop taking some or all of their regular drugs if electrocorticography is utilized as an exception. Before surgery, a complete blood count, renal function test, and liver function tests must be evaluated. Long-term use of anti-epileptic drugs might cause thrombocytopenia, leucopenia, and abnormal liver function tests [12].

On the morning before surgery, anti-convulsants should be continued and premedicated with benzodiazepines, atropine, or glycopyrrolate. Electrocardiography, pulse oximetry, blood pressure, and urine output are essential to intraoperative monitoring. The guidelines for establishing and maintaining an adequate intravenous anesthetic drug concentration in the plasma and brain, the elements defining the right target drug concentration to strive for, and the methods for adjusting this in response to the patient's reaction are among the knowledge necessary for an anesthetist using TIVA. Practical considerations in ensuring that the patient receives the correct medication dosage and monitoring TIVA patients is essential. Maintaining proper cerebral perfusion pressure and avoiding a rise in intracranial pressure are among the fundamentals of neuroanesthesia [2]. It is preferable to avoid nitrous oxide (N_2_O) since it causes an increase in cerebral blood volume, thus leading to a rise in cerebral metabolic rate. It is complicated how inhalation anesthetics affect cerebral blood flow; a rise in cerebral blood volume is typically counterbalanced by a fall in cerebral metabolic rate [12]. Inhalational anesthesia is avoided in patients with malignant hyperthermia, in those at risk of post-operative nausea and vomiting, and when intra-operative monitoring of somatosensory or motor-evoked potentials is required [2]. In animal models (cats), nitrous oxide (N_2_O) causes seizures. However, this has yet to be observed in people. After brief exposures to N_2_O, mice have displayed withdrawal seizures [13]. In a pressure chamber under the anesthesia of 1.55 atm absolute N_2_O, participants demonstrated stiffness of the muscles with jerky movements, laborious and quick breathing, perspiration, and dilated pupils [14].

## Conclusions

This case report shows that TIVA may be a safe and effective therapy for conscious sedation, preventing and controlling refractory seizures, notably when other failed treatments. Many medications used have convulsant and anti-convulsant properties, which impact the anesthetic choice. More research is required to evaluate the efficacy and safety of TIVA for seizure patients, and based on each patient's specific condition, the use of TIVA should be carefully monitored and individualized. Studies have shown that TIVA can provide better seizure control and reduce the risk of adverse events compared to other anesthesia methods, such as inhalational anesthesia. TIVA is also associated with fewer side effects, such as nausea and vomiting, and has a quicker recovery time.

It is crucial to remember that not all patients may benefit from TIVA, and other aspects like patient age, medical history, and the type of seizure being treated should be considered when choosing the best anesthesia strategy. Overall, TIVA is a valuable option for managing sedation and seizures and can be a safe and effective alternative to other anesthesia methods for appropriate patients.
